# New biotechnological perspectives of a NADH oxidase variant from *Thermus thermophilus *HB27 as NAD^+^-recycling enzyme

**DOI:** 10.1186/1472-6750-11-101

**Published:** 2011-11-03

**Authors:** Javier Rocha-Martín, Daniel Vega, Juan M Bolivar, Cesar A Godoy, Aurelio Hidalgo, José Berenguer, José M Guisán, Fernando López-Gallego

**Affiliations:** 1 Departamento de Biocatálisis. Instituto de Catálisis y Petroleoquímica-CSIC. Campus UAM. Cantoblanco. 28049 Madrid, Spain; 2 Centro de Biología Molecular Severo Ochoa. CSIC-UAM. Departamento de Biología Molecular. Campus UAM. Cantoblanco. 28049 Madrid, Spain

**Keywords:** NAD+, extremophiles, dehydrogenase, immobilization

## Abstract

**Background:**

The number of biotransformations that use nicotinamide recycling systems is exponentially growing. For this reason one of the current challenges in biocatalysis is to develop and optimize more simple and efficient cofactor recycling systems. One promising approach to regenerate NAD^+ ^pools is the use of NADH-oxidases that reduce oxygen to hydrogen peroxide while oxidizing NADH to NAD^+^. This class of enzymes may be applied to asymmetric reduction of prochiral substrates in order to obtain enantiopure compounds.

**Results:**

The NADH-oxidase (NOX) presented here is a flavoenzyme which needs exogenous FAD or FMN to reach its maximum velocity. Interestingly, this enzyme is 6-fold hyperactivated by incubation at high temperatures (80°C) under limiting concentrations of flavin cofactor, a change that remains stable even at low temperatures (37°C). The hyperactivated form presented a high specific activity (37.5 U/mg) at low temperatures despite isolation from a thermophile source. Immobilization of NOX onto agarose activated with glyoxyl groups yielded the most stable enzyme preparation (6-fold more stable than the hyperactivated soluble enzyme). The immobilized derivative was able to be reactivated under physiological conditions after inactivation by high solvent concentrations. The inactivation/reactivation cycle could be repeated at least three times, recovering full NOX activity in all cases after the reactivation step. This immobilized catalyst is presented as a recycling partner for a thermophile alcohol dehydrogenase in order to perform the kinetic resolution secondary alcohols.

**Conclusion:**

We have designed, developed and characterized a heterogeneous and robust biocatalyst which has been used as recycling partner in the kinetic resolution of *rac*-1-phenylethanol. The high stability along with its capability to be reactivated makes this biocatalyst highly re-useable for cofactor recycling in redox biotransformations.

## Background

Dehydrogenases catalyze a great variety of redox reactions in fine chemistry (asymmetric reduction of pro-chiral acetones, selective oxidations of polyols....) [1-5]. However, since they require nicotinamide cofactors to catalyze substrate reduction or oxidation, their biotechnological implementation must address the issues of cofactor stability and thermodynamic equilibria that otherwise prevent quantitative substrate conversion [6-9]. Cofactor recycling *via *electrochemical, photochemical or enzymatic methods is one alternative to overcome these issues [10]. Enzyme-mediated cofactor recycling is one of the most promising approaches to address redox reaction limitations, enabling quantitative substrate conversions [10,11]. The number of biotransformations that uses nicotinamide recycling systems is exponentially growing. For this reason one of the current challenges in biocatalysis is to develop and optimize more simple and efficient cofactor recycling systems [6-9,12].

One way to recycle NAD^+ ^is the enzyme-mediated oxidation of the corresponding reduced cofactor using molecular oxygen as an oxidizing agent. In nature, there are two types of NADH-oxidases (EC 1.6.3.1) depending on their catalytic mechanism: 1) enzymes that oxidize NADH through the two-electron reduction of hydrogen peroxide to two molecules of water [13,14], and 2) enzymes that catalyze the oxidation of NADH by reducing molecular oxygen to hydrogen peroxide [15-17]. Water-forming NADH-oxidases are more interesting for biotechnological applications due to the innocuous nature of water as a byproduct. Conversely, the recent application of catalases for *in-situ *elimination of hydrogen peroxide [18] would boost the application of H_2_O_2_-forming NADH-oxidases in biocatalysis.

The H_2_O_2_-forming NADH-oxidases are flavoenzymes, where the flavin cofactor acts as electron mediator, carrying the electrons from NADH to molecular oxygen [15,16,19]. In the last two decades, many H_2_O_2_-forming NADH-oxidases have been isolated and characterized from both mesophilic and thermophilic organisms [15-17,19]. Enzymes from thermophilic microorganisms are interesting biocatalysts, because their thermostability is much higher than those from mesophilic origin [20,21]. Such resistance to high temperatures facilitates their purification by thermal shocks when they are overproduced in mesophilic hosts like *E. coli*.

The enormous biotechnological potential of these enzymes have encouraged biotechnologists to approach different downstream strategies to fulfil the stability and productivity requirements imposed by the industry to the enzyme catalysts. Immobilization is presented as a useful technology for simultaneously overcoming two primary industrial limitations: re-using and stability [22-25]. For the last 50 years many immobilization protocols have been successfully applied to enzymes [26-29]. Immobilization techniques may promote enzyme stabilizations that would increase the life-time of the catalyst and therefore the potential of the enzymes as industrial catalysts [30-35]. Recently, re-using of immobilized catalysts has been described through reactivation of inactivated insoluble preparations of a survey of enzymes [36-38]. Therefore, merging of immobilization and reactivation technologies would be able to multiply the biocatalyst lifetime.

We report the isolation, purification and characterization of a NADH-oxidase from *Thermus thermophilus *HB27 and its preliminary optimization for biotechnological purposes. This enzyme albeit 99% identical to that found in *Thermus thermophilus *HB8, presented relevant biochemical differences that encouraged us to study some of its biochemical and biotechnological features for its application in cofactor regeneration.

## Results

### Isolation and expression of recombinant NOX

The gene TTC0057 was amplified from genomic DNA of *Thermus thermophilus *HB27 as described in Materials and Methods. The sequence of the cloned gene revealed a tyrosine at position 194 as opposed to a histidine found at the same position in the published genome of *T. thermophilus *HB27 [39]. This difference was corroborated through a second amplification, cloning and sequencing of the gene from the genomic DNA, suggesting that either the published sequence contained an error or that our strain had acquired a mutation during its growth and maintenance in the laboratory over the years. It is worth to note that such His was also found at position 194 in the well-studied and 99% identical enzyme from *T. thermophilus *HB8 [40] for which the 3D structure is available (PDB code 1NOX). To characterize this variant and shed light on the effect of this single mutation on its activity, the amplified TTC0057 gene was cloned into a pET22b expression vector to overexpress the protein in *E. coli *BL21. The vast majority of the recombinant protein was obtained in the soluble fraction facilitating its purification (Additional file 1 Figure S1).

### Temperature-based purification

Since this enzyme is from a thermophilic microorganism but cloned in a mesophilic one, purification through thermal shock was approached as the simplest way to achieve high purification factors [41]. Crude extract from *E.coli *containing NOX was incubated at 80°C, achieving a purification factor of 7.5 with a yield of 100% (Table 1 and Additional file 1 Figure S1 (SDS-PAGE)).

**Table 1:** Purification of NADH oxidase from *E.coli*.

Entry^a^	Protein (mg/ml)	Specific Activity (U/mg)^b^	Purification factor	Purification yield (%)
**1**	26.29	0.25	1	100

**2**	6.48	1.88	7.5	100

**3**	3.3	3.69	14.7	80

**4**	2.93	4.15	16.6	70

**^a^**Entry 1: Crude extract. Entry 2: Heat treatment (80°C) for 45 min. Entry 3: Heat treatment (80°C 45 minutes) and further incubation with PEI-ag for 30 min. Entry 4: Heat treatment (80°C 45 minutes) and sequential **^b ^**activity at 65°C, pH 7 without exogenous FAD/FMN.

In order to completely purify NOX, ionic chromatographic steps were further carried out using two different matrixes (polyethylenimine agarose beads (PEI-ag) and sulfate-dextran agarose beads (SD-ag)). These two polymeric coated ionic exchangers are able to absorb the majority of proteins from an *E. coli *extract [33,35]. Notably, neither PEI-ag nor SD-ag bound NOX, while other proteins form the crude extract were bound to both resin. Consequently, NOX was ever purer at the supernatant fraction after such ionic step. The designed purification protocol was: a thermal treatment at 80°C for 45 minutes, followed by incubation of the supernatant with PEI-ag (purification factor improved two fold) and then with SD-ag. The final purification protocol gave a yield of 70% with a purification factor higher than 16 (Table 1 and Additional file 1 Figure S1).

### Biochemical characterization of purified NOX

#### NOX is flavin-dependent and H_2_O_2_-forming oxidase

**Figure 1:**
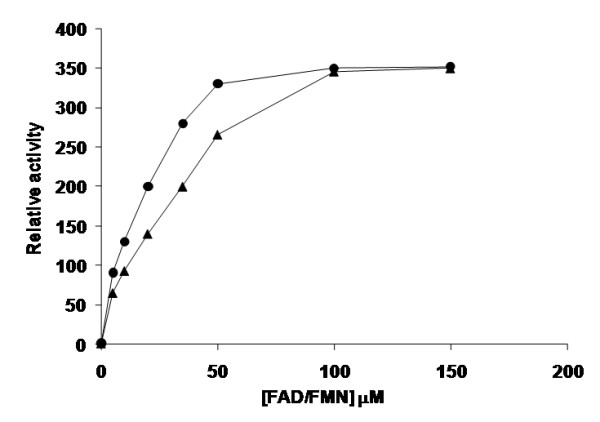
**Effect of exogenous flavin cofactor on the activities of recombinant NOX in soluble form**. Before purification, crude extracts were incubated with different flavin nucleotides at different concentrations (from 0 to 150 μM of either FMN or FAD). Activity was determined in 50 mM sodium phosphate at pH 7 and 37°C. Soluble enzyme with FAD (*triangles*), soluble enzyme with FMN (*circles*). The relative activity was calculated taking as fraction 1 the initial observed activity without flavin cofactor.

The pure enzyme is a monomer with an electrophoretic mobility corresponding to the size ≈27 KDa, as expected from its sequence. It is able to oxidize NADH to NAD^+ ^reducing equimolar amounts of oxygen [15,17,19]. (Additional file 2 Table S1). The crude extract was incubated with different flavin nucleotides. The NOX activity was rather low in the absence of externally-added flavin nucleotide. However, when the cofactor was exogenously added, the activity increased at higher flavin cofactor concentrations up to a maximum activity at 100 μM of flavin mono- or di-nucleotide (Figure 1). This dependence on flavin cofactor addition confirms that the flavin cofactor is not covalently bound to the native enzyme, as opposed to other oxidases [19,42].

#### Kinetic parameters

NOX is quite active at low temperatures (25-37°C) relative to other enzymes from thermophilic sources. For this reason, steady-state kinetic parameters of this enzyme were calculated at 25°C for the flavin mono- and di-nucleotide cofactors and for the NADH (Table 2). Interestingly, the NOX (HB27) in presence of exogenous flavin cofactor (150 μM) showed 6-fold higher catalytic efficiency towards NADH compared to its counterpart NOX (HB8) under the same conditions. This significant difference was due to the lower *Km *and higher *kcat *values in favour of the enzyme from our HB27 strain and it may be explained by three amino acids found in the primary sequence of the HB27 strain (K166, H174 and Y194) that differ from those found in the HB8 strain (R166, R174 and H194)

**Table 2:** Kinetics parameters of NOX towards different cofactors.

	*Km* *(*mM)	*kcat* *(s-1)*	*kcat/Km* × 10^6^(M-1*s-1)
NADH^a^	2.1 ± 0.4	15.6 ± 0.7	7.4

FAD^b^	34.1 ± 6.0	-	-

FMN^b^	42.8 ± 5.1	-	-

The steady-state kinetics parameters were calculated at pH 7 and 25°C (See methods). Activities were adjusted to a non-linear regression.**^a ^**Kinetics parameters for NADH were calculated using 50 μM FAD.**^b ^**Kinetics parameters for flavin cofactors were calculated using 10 μM NADH.

#### Temperature and pH profiles

**Figure 2:**
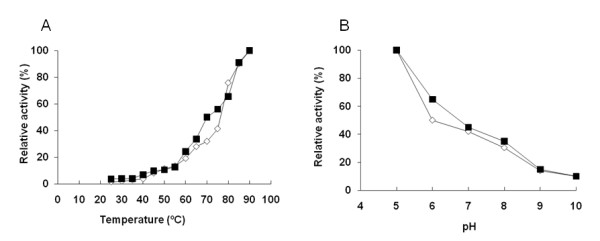
**Influence of temperature (A) and pH (B) on the enzymatic activity of soluble and immobilized NOX forms**. In the case of the temperature, activity assays were performed in 50 mM sodium phosphate at pH 7, FAD 150 μM and temperatures ranging 25-90°C. On the contrary, pH effect on activity was analyzed keeping constant the temperature at 65°C at different pH values, ranging between 5-9, in presence of FAD 150 μM. In both experiments, soluble (*empty rhombus*) and immobilized (on CNBr-agarose) NOX (*full squares*) preparations were analyzed. For both graphs, relative activity was calculated assigning 100% of activity to the highest measured activity at one particular temperature and pH.

Other important parameters used to evaluate the biotechnological potential of an enzyme are its response to broad range of pH and temperature, as operational conditions often vary from physiological ones. Moreover, incubation of enzymes at extreme conditions may trigger enzyme aggregation or precipitation. We have immobilized NOX on agarose activated with cyanogen bromide (CNBr-ag) to softly attach the protein to the support through a covalent bond, in an attempt to avoid protein aggregation but without pursuing the protein stabilization by immobilization. Under mild conditions (see methods) NOX was quantitatively immobilized onto CNBr-ag, recovering 80% of the immobilized activity. Both soluble and insoluble enzyme preparations behaved quite similarly under a broad range of pH and temperatures, indicating that no aggregative effects were taking place (Figure 2). We were unable to measure enzyme activity at temperatures above 90°C due to technical problems as cofactor stability (Figure 2A). Therefore, 90°C was the temperature where the highest activity was measured, confirming that this enzyme was extremely active under high temperatures. On the other hand, this enzyme as well as its counterpart from HB8 strain presented an acidic optimal pH (pH 5) (Figure 2B)[19], pH values lower than 5 could not be measured because NADH was unstable under those conditions.

#### Temperature induces NOX hyperactivation at low flavin cofactor concentrations

**Figure 3:**
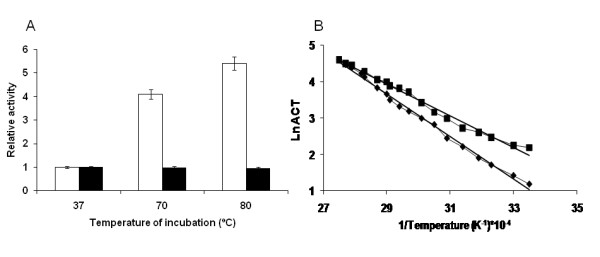
**NOX hyperactivation induced by thermal incubation**. **(A) **NOX was incubated at different temperatures (37, 70 and 80°C) for 1 h and then measured at 65°C, in 50 mM sodium phosphate at pH 7 under either limited (white bars) or saturated (black bars) flavin cofactor conditions (150 μM). The relative activity was calculated for each condition (limited or saturated flavin cofactor), taking as fraction 1 the initial activity observed without thermal incubation. **(B) **Arrhenius' plot of the reaction catalyzed by soluble NOX which was thermal incubated at 80°C for 45 minutes (*squares*) and by soluble NOX which did not undergo to thermal incubation (*rhombus*). The negative linear regressions were calculated for each sample, resulting the following equations: for thermally incubated NOX (y = -4339,9 × + 16,52. *R^2 ^= 0,987*) and for non thermally incubated NOX (y = -5828,8 × + 20,571. *R^2 ^= 0,995*).

As it has been mentioned previously, exogenous flavin cofactor was needed to achieve high enzymatic activities. However, when no flavin cofactor was added, an unexpected effect was observed when NOX was incubated at high temperatures. Incubated enzymes were up to 6-fold more active than those which remained at 37°C. In fact, the higher the temperature of incubation, the higher the hyperactivation achieved (Figure 3A). This result was confirmed by analysis of the Arrhenius' plots (Figure 3B). It is evident that the enzyme incubated at high temperature presented lower activation energy than those which did not (36 ± 1 *versus *48 ± 0.8 KJ/mol). The thermal-dependent hyperactivation drove the enzyme to a relatively high specific activity (4.5 U/mg) in limited flavin cofactor conditions (no exogeneous cofactor was added) at 37°C, which was 8 times lower than the activity in presence of that flavin cofactor (37.5 U/mg) under the same conditions.

### Stabilization of NOX *via *immobilization

**Figure 4:**
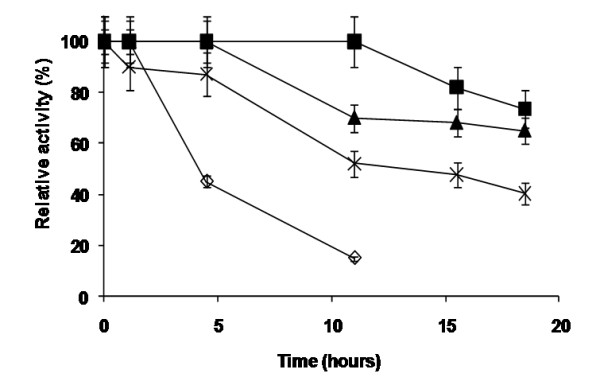
**Thermal stability of different preparation of NOX immobilized onto differently activated agarose surfaces**. The preparation of different NOX insoluble derivatives was carried out according to that described in Methods. 8 U (at 65°C) of each NOX insoluble preparations were incubated in 50 mM sodium phosphate at pH 7 and 83°C. At such conditions, half-life time of soluble NOX can be quantified in less than 24 h, allowing thus to calculate the stabilization factors achieved by each immobilization protocol. Stabilization factor was defined as the ration between the half-life times of each immobilized derivative and the soluble preparation. NOX preparations studied here were; soluble (*empty rhombus*), immobilized onto IDA-Cu^2+^-ag (*crosses*), immobilized onto CNBr-ag (*full triangles*) or immobilized onto Gx-ag (*full squares*). In all cases the crosslinking percentage of agarose was 6% (agarose-6BCL). Results represents the mean (± SD) of three different experiments.

Protein immobilization has been shown as an interesting alternative to overcome two important hurdles that enzymes are faced with in order to be used on an industrial scale: Re-using and stability. To this end, we have immobilized NOX onto agarose through three different chemistries. Firstly, NOX was covalently attached to CNBr-ag *via *its N-terminal [43]. This immobilization was performed under very mild conditions to have an enzyme preparation with properties very similar to those of the soluble enzyme [44,45], but where enzyme-enzyme interactions were diminished. Secondly, NOX was reversibly immobilized *via *IMAC chemistry onto agarose activated with metal chelates (Cu^2+^) (IDA-Cu^2+^-ag) [46,47]. In the resulting enzyme-agarose complex (IDA-Cu^2+^-NOX), the enzyme was immobilized and properly oriented using native histidine rich regions [47]. Finally, NOX was immobilized at alkaline pH values onto agarose activated with glyoxyl groups (Gx-ag) where the enzyme was immobilized through its lysine rich regions, resulting in very intense covalent attachments [43]. Immobilization yields and recovered activities depended on the respective immobilization protocol (Table 3). Gentle covalent immobilization on CNBr-ag and reversible immobilization on IDA-Cu^2+^-ag, resulted in 100% of immobilization yield and 80% of expressed activity. Conversely, NOX was not quantitatively immobilized on on Gx-ag, and around 40% of the enzymatic activity was lost during the immobilization process (Table 3). When thermal stability was analyzed for each insoluble derivative, the immobilized enzyme always showed an increase in enzyme stability relative to soluble preparations. Immobilization on Gx-ag matrixes yielded the most stable NOX preparation. Contrarily, the non-covalent immobilization via metal-chelate binding in IMAC-material led to low stabilization factors, even lower than the gentle covalent immobilization on CNBr-ag (Figure 4).

**Table 3:** Parameters of NOX immobilization onto agarose via different chemistries.

Activated agarose^a^	Immobilization yield Ψ (%)^b^	Expressed activity Ae (%)^c^
**Gx-ag^a^**	85	60

**CNBr-ag^a^**	100	80

**IDA-Cu^2+^-ag^a^**	100	80

**^a ^**6BCL agarose was activated with different functional groups as methods described in order to immobilized NOX through different chemistries, giving each one different properties to the final insoluble preparation. **^b ^**Immobilization yield was calculated as follows; Ψ (%) = (Supernatant activity after incubation with the support/Supernantant activity before incubation with the support)*100.**^c ^**Expressed activity was calculated; Ae (%) = (Activity/g of support)/(Immobilized activity/g of support)*100.

### Solid-phase biocatalyst re-activation

**Figure 5:**
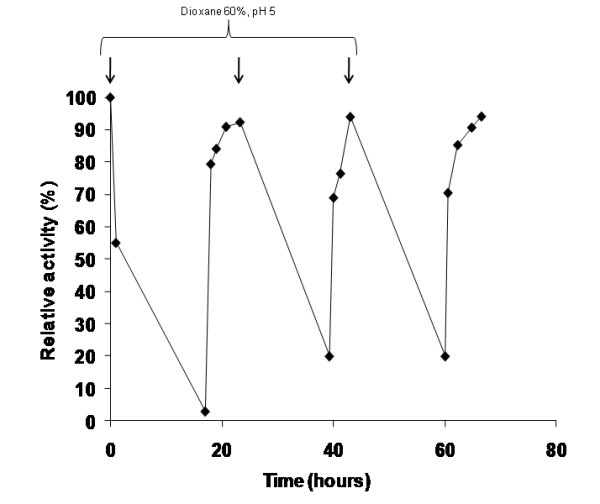
**Reactivation of Gx-NOX catalyst after inactivation induced by organic solvents**. Gx-NOX (*rhombus*) was incubated with 60 vol% of dioxane in 10 mM sodium acetate buffer at pH 5 and 37°C for 18 h. The inactivated preparation was vacuum filtered and then reactivated by incubation in 10 mM sodium phosphate buffer at pH 7 and 65°C. The same protocol was repeated up to three times, the inactivation step was depicted on the plot as an arrow. Different samples of both inactivation and reactivation steps were withdrawn in order to analyze enzyme activity as described in Methods. The activity of different inactivation/reactivation cycles was normalized assigning 100% of relative activity to the initial activity of biocatalyst right before being inactivated for the first time.

Recently, our group has developed new strategies for the reactivation of industrially relevant enzymes immobilized by covalent attachment [36,37]. The ability to reactivate biocatalysts for use in additional operation cycles has provided a new avenue of research focused on biocatalyst reuse on an industrial scale. Since Gx-NOX was the most stable derivative, that preparation was subject to different inactivation/reactivation cycles. Inactivation was carried out under high dioxane concentration (60 vol%), weak acidic pH (pH 5) and 37°C, mimicking a harsh set of conditions where all enzyme activity was lost, even in the case of the stabilized derivative. The inactivated Gx-NOX was incubated in sodium phosphate buffer at pH 7 for several hours in order to recover the biocatalyst's initial activity. This insoluble derivative fully recovered its initial activity in less than 8 h, and reactivation was found to be quantitatively effective for at least three cycles (Figure 5). The reactivation of NOX was more efficient when the enzyme was immobilized on Gx-ag, due in large part to the rigidity and robustness provided by the immobilization chemistry.

### Kinetic resolution of *rac*-1-phenylethanol

**Figure 6:**
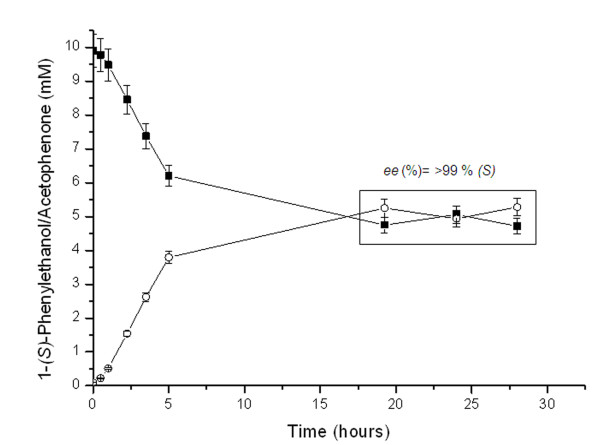
**Reaction course of the kinetic resolution of *rac-*1-phenyl ethanol**. Reaction was performed using a NAD+ regeneration system consisting of 0.2 mg of NOX immobilized on Gx-ag as method described and 4 mg of alcohol dehydrogenase from *Thermus thermophilus *HB27 immobilized as described elsewhere [41]. The reaction mixture contained 0.5 mM NADH, 7.5 mM rac-1-phenylethanol in 10 mM sodium phosphate at pH 7 and 45 C. Substrate conversion was determined by HPLC as previously described [41]. In the same way, enantiomeric excess (ee) at 50% of conversion was determined by chiral HPLC as previously described [41]. Symbols: acetophenone (*empty circles) *and 1-(S)-phenylethanol (*full squares*).

NOX was used as re-cycling partner of a secondary alcohol dehydrogenase from *Thermus thermophilus *(TtADH), for the kinetic resolution of *rac*-1-phenylethanol at 55°C and pH 7. TtADH enantioselectively oxidizes 1-(*S)*-phenylethanol to acetophenone [41], reducing NAD^+ ^to NADH. In Figure 6 is shown how the bi-enzymatic system was able to oxidize the substrate reaching 99% of enantiomeric excess of 1-(*R)*-phenylethanol at 50% of conversion. The cofactor re-cycling system has allowed the addition of only 6.5% mol of NADH relative to substrate. The total turnover number of NADH was 10 in each reaction cycle, however it is worth noting that this value could be increased either by decreasing the cofactor concentration or by increasing the substrate concentration. In terms of productivity, 1 mg of immobilized NOX on Gx-Ag was able to recycle 40 μmol of NAD^+ ^per hour under the conditions described here. The same result was observed when the reaction was carried out with the reactivated NOX derivative. Herein we have validated a multi-enzyme system which may be used for kinetic resolutions of a wide variety of secondary alcohols.

## 4. Discussion

We have purified and characterized a variant NADH-oxidase from *Thermus thermophilus *HB27. This enzyme oxidizes NADH to NAD^+^, utilising oxygen as an electron acceptor and a flavin cofactor as an electron mediator, to yield hydrogen peroxide as a product. The amino acid sequence of this NOX enzyme presents 98.5% identity to a similar enzyme isolated from *T. thermophilus *HB8. However, the new NOX variant presented 5.9-fold higher catalytic efficiency than its counterpart from the strain HB8, a difference that is likely related to the three divergent residues found at positions 166, 174 and 194 (Additional file 3 Figure S2). Noteworthy, the reported sequence for NOX also differs from the sequence published for the HB27 strain by a single amino acid change at position 194 (H194Y), suggesting either an annotation error at the original sequence [39] or an adaptation of the strain to the laboratory conditions. Because samples of the HB27 strain obtained from other laboratories did not contain such mutation, it is tempting to speculate that higher NADH-oxidase activity might enhance bacterial fitness in the very rich TB medium routinely used in the laboratory, where an excess of reducing power (increased NADH/NAD^+ ^ratio) is likely to occur. In any case, we have found a potential target on NOX primary sequence to further optimize its catalytic performance by protein engineering.

Steady-state kinetic parameters were calculated for purified NOX with three different cofactors, NADH, FMN and FAD. Analyses of the results, revealed one main difference between this NOX and that from the HB8 strain, the catalytic efficiency of NOX towards NADH was higher than what it was reported for that one from the HB8 strain. Currently, mutagenesis experiments are being undertaken in order to shed light on the specific role of the three residues that differ between both proteins on the catalytic activity.

We also described practical thermal hyperactivation of this enzyme under limited conditions of flavin cofactor. This insight suggests a more active NOX conformation at high temperatures, resulting in higher affinity for the flavin cofactor. This beneficial conformation and associated good specific activity can be fixed and retained in further downstream enzyme applications even at low temperature. The nature of this thermally induced conformational change is not clear at present. We suggest that it could be due to enhanced binding of the flavin cofactor, since it was only observed under conditions in which the flavin cofactor is limiting. Similar hyperactivation effects after high temperature incubation have been described for other thermophilic proteins also likely due to conformational reordering of particular regions of the protein missfolded during expression at low temperatures [48-50]. Whatever the reason, this feature gives the NOX described here a high potential for industrial use in NADH recycling in redox reactions. In this sense, we have shown its use coupled to a dehydrogenase for alcohol oxidations at mild temperatures without a requirement for exogenous addition of flavin cofactor, making the process much more cost-effective.

Thinking on these applications, we immobilized the NOX in a broad number of surfaces. In the literature, different immobilization protocols have been used for the immobilization of NADH oxidases especially in the biosensors field [51-53]. From our experiments, covalent attachment of NOX to an agarose support activated with glyoxyl groups resulted, to the best of our knowledge, in the most thermostable NADH-oxidase preparation reported so far. This Gx-NOX derivative shows at the same time irreversible binding of the enzyme and a likely homogeneous protein orientation because preferential covalent binding occurs through the lysine richest regions [31]. These properties related to the immobilization chemistry are the basis for the successfully application to a large number of enzymes, all of which achieved a high stabilization factors [31].

In addition to the high stability of the insoluble biocatalyst, NOX can also be successfully reactivated once it has been fully or partially inactivated. Here, we have presented the first report of protein reactivation for immobilized NOX on highly stabilized derivatives. Moreover, these derivatives can be efficiently reactivated several times by incubation on phosphate buffer after inactivation cycles in 60 vol % dioxane. An explanation for this could be based on the fact that organic solvents drive enzyme to local distortion rather than a global unfolding [54]. These local distortions may be located on the surface of the enzyme because organic solvents may strip the external water layer essential for enzyme activity [55-57] (easier to recover when organic solvent is removed) or might occur at a higher scale by unfolding internal domains of the proteins because penetration of the solvent to the hydrophobic core (more difficult to revert). As multipoint covalent immobilization limits severe unfolding [58], the enzyme can be easily reactivated simply by elimination of the organic solvent. The same behavior was found for other enzymes like lipases [37] and even for other thermophilic oxidoreductases such as glutamate dehydrogenase from *Thermus thermophilus *HB7 [36]. In this last example, recovery of active quaternary structure was achieved, making a breakthrough in solid-phase enzyme reactivation protocols for multimeric enzymes.

Finally, this highly stable and heterogeneous biocatalyst was applied as cofactor recycling partner of a main alcohol dehydrogenase from the same thermophilic source, to kinetically resolve pharmaceutically relevant compounds such as 1-phenylethanol. Expectedly, the selectivity of the main alcohol dehydrogenase was extremely high [59] and cofactor recycling by NOX allowed reaching the maximum yield using only 6.5% mol of cofactor, which means that one mol of cofactor was recycled up to 10 times per reaction cycle under the conditions studied here. Moreover, since this heterogeneous biocatalyst is highly stable and can be re-used for many cycles, the total turnover of such catalyst can be increased. In addition, and still to be tested, coupling of a catalase to the system should improve the global stability of the enzymes involved in the process. The catalase would be able to, *in-situ*, eliminate the hydrogen peroxide formed as byproduct by NOX. As a result there would be decreased accumulation of such compound capable of inactivating the protein biocatalysts, which would lead to further optimization of both conversion rate and final yields.

## Conclusion

The high re-usability of immobilized NOX encouraged us to develop new bi-enzymatic systems where NAD^+ ^recycling is needed. The thermophilic nature of NOX and its ability to be reactivated make feasible the use of this catalyst in redox biotransformation coupled to H_2_O_2 _removal systems, p.e catalase. This catalyst has been successfully applied as re-cycling system to the kinetic resolution of secondary alcohols at high temperatures. Further, protein engineering studies need to be done to improve NADH-oxidase performance at lower temperatures, in order to couple it to mesophilic alcohol dehydrogenases.

## Methods

### Materials

Nicotinamide adenine dinucleotide (NADH) was purchased from Jülich Fine Chemicals (Codexis, Redwood city, CA). Flavin adenine dinucleotide (FAD), flavin mononucleotide (FMN), polyethyleneimenine (PEI) (MW: 600-1000 kDa) and sulfate-dextran (MW: 100 kDa) were supplied by Sigma-Aldrich Co (St. Louis, IL). Iminodiacetic acid disodium salt monohydrate (IDA) and copper sulphate (II) 5-hydrate were purchased from Fluka (Buchs, Switzerland). Cyanogen bromide 4B Sepharose and crosslinked agarose beads (4%) were from GE Healthcare (Uppsala, Sweden). Polyethyleneimine agarose beads (PEI-ag) supports were prepared as previously described elsewhere [33]. IMAC supports Glyoxyl agarose beads (Gx-ag) and Sulfate-dextran agarose beads (SD-ag) were prepared as previously described [46,60,35]. Protein concentration was determined using the method of Bradford [61]. All other used reagents were of analytical grade.

### Cloning and expression of the NOX

#### Bacterial strain, plasmids and growth conditions

*Thermus thermophilus *HB27 used as DNA source for NOX cloning was a laboratory-adapted strain derived from the original strain donated by Prof Koyama [62]. *E. coli *strains DH5α [*sup*E44, Δ*lac*U169 (Δ80 *lac*ZΔM15), *hsd*R17, *rec*A, *end*A1, *gyr*A96, *thi-1, relA1*] and BL21 DE3 [*hsd*S, *gal *(Δ*c*Its857, *ind*1, *Sam7, nin5, lacUV5*-T7 gene 1)] were used for cloning and expression purposes, respectively. The thermophile was grown at 70°C in TB (*Thermus *Broth) [63] under stirring (150 r.p.m.) and *E. coli *was grown at 37°C in modified Luria-Bertani (LB) medium [64]. Ampicillin (100 mg/L) or kanamycin (30 mg/L) were added to the cultures when required for selection.

#### Plasmid construction

DNA isolation, plasmid purification, restriction analysis, plasmid construction and DNA sequencing were carried out by standard methods[65]. The Polymerase Chain Reaction (PCR) was performed using a mixture of *Tth *and *Pfu *DNA polymerases as described by the manufacturer (BIOTOOLS B & M, Madrid, Spain). For the construction of the expression vector, the gene TTC0057 coding for the NOX enzyme was amplified using the primers TTC0057 - *Nde*I Forward: (5'-TTCCATATGATGGAGGCGACCCTTCC-3') and TTC0057 - *Eco*RI Reverse: (5'-TTCGAATTCCTAGCGCCAGAGGACCAC-3'), which included restrictions sites for *Nde*I and *Eco*RI (underlined). The PCR product was subsequently cloned using the same sites into the expression vector pET22b+, (pET22b-TTC0057). The cloned gene was sequenced by standard methods.

### Expression of the recombinant protein in *E. coli*

Plasmid pET22b-TTC0057 was transformed into the expression strain *E. coli *BL21(DE3), which carries the RNA polymerase gene from the T7 phage under the control of an inducible promoter. The transformed cells were grown at 37°C in of LB with ampicillin until the culture reached an optical density of 0.6 at 600 nm. Then, the expression of the T7 RNA polymerase was induced by addition of iso-propyl-1-thio-β-D-galactopyranoside (IPTG) to a concentration of 1 mM. The bacterial culture was incubated at 37°C for further 2 h, and cells were harvested and washed in sodium phosphate buffer by centrifugation (10000 × g, 10 min) before being stored as wet pellets at -20°C until use.

### Determination of enzyme activity and kinetics parameters

The activities of the different NOX preparations were analyzed by following the decrease in absorbance at 340 nm corresponding to the oxidation of NADH. A sample of the enzyme preparation (10-100 μL) was added to a spectrophotometer cuvette containing 2 mL of 50 mM sodium phosphate buffer at pH 7 and 37°C and 50 μL of 10 mM NADH was added. When indicated, different temperatures and pH values were used. One activity unit (U) was defined as the amount of enzyme required to oxidize 1 micromol of NADH per minute at pH 7 at the indicated temperature (standard activity is given at 25°C, 37°C or 65°C). In all cases, the pH value was adjusted at the indicated temperature using a pH-meter with temperature sensor.

Kinetic parameters were calculated from the initial velocity of NADH consumption assays. The reaction was initiated by adding NOX to the reaction mixture. Measurements were performed at 37°C in 50 mM sodium phosphate at pH 7, at different NADH concentrations. Assays were performed in triplicates at each concentration. Results were fitted using the Michaelis-Menten equation based non-linear regression analysis of the data at each fixed concentration [66].

### Purification of the NOX

Cells were lysed by sonication, and the cell debris was eliminated by centrifugation (10,000 × g for 10 min). Crude protein extracts were diluted 10 fold in 10 mM sodium phosphate at pH 7 and incubated at 80°C and pH 7 for 45 min. Protein aggregates were discarded after centrifugation (10,000 × g for 10 min), and the clarified supernatant containing the NOX activity was offered to different chromatographic supports (PEI-ag and SD-ag) at pH 7 and 25°C (1 g of support per 10 mL of protein extracts). Periodically, the activity of NOX and the concentration of proteins were analyzed in both the supernatant and the suspension fractions to monitor the purification process.

### Enzyme immobilization on CNBr-activated sepharose 4 BCL

The immobilization was carried out by adding 2 g of the CNBr-activated support to 20 mL of 10 mM sodium phosphate at pH 7 containing 8 U (at 65°C) of NOX. The suspension was kept under mild stirring for 15 minutes at 4°C. Afterward, the support was filtered and washed with 10 mM sodium phosphate buffer at pH 7 and incubated for 2 hours in 1 M ethanolamine at pH 8 to block the remaining reactive groups. Finally, the immobilized preparation was washed with 10 mM sodium phosphate at pH 7.

### Enzyme immobilization on agarose activated with metal chelates

A volume of 20 mL of 10 mM sodium phosphate at pH 7 containing 8 U (at 65°C) of NOX was mixed with 2 g of metal-IDA support. The suspension was gently stirred at 25°C. Samples of both supernatant and suspension were withdrawn at different times to analyze enzyme activity as described above. Finally, the immobilized preparation was washed with 10 mM sodium phosphate at pH 7.

### Enzyme immobilization on agarose activated with glyoxyl groups

2 g of activated agarose were incubated with 20 ml of 100 mM sodium bicarbonate pH 10.05 containing 8 U (at 65°C) of soluble NOX. The suspension was gently stirred at 25°C. Samples of both supernatant and suspension were withdrawn at different times to analyze enzyme activity as described above. Finally, the immobilized preparations were reduced for 30 minutes at 25°C with 20 mg sodium borohydride as described elsewhere [31]. After this period the preparation was washed with an excess of 10 mM sodium phosphate at pH 7 and assayed.

### Effects of temperature and pH on the activity of soluble enzyme and immobilized NOX

The activities of soluble and CNBr-NOX preparations were assayed at different temperatures (from 25 to 90°C) in 50 mM sodium phosphate and at different pH values (pH 5-10). The following buffer systems were used (50 mM): sodium acetate (pH 5.0), sodium citrate (pH 6.0), sodium phosphate (pH 7.0 and pH 8.0) and sodium carbonate (pH 9.0 and pH 10.0). All pH values were adjusted at 65°C using a pH-meter with temperature sensor.

### Thermal inactivation assays

Different NOX preparations (soluble and immobilized) were incubated in 50 mM sodium phosphate at pH 7 and 83°C. Samples were withdrawn at different times and their activity was measured as previously described.

### Chemical Inactivation/Reactivation cycles (Reactivation experiments)

Gx-NOX was incubated with 60 vol % of dioxane in 50 mM sodium acetate buffer at 37°C and pH 5 for 18 h. The inactivated preparation was vacuum filtered and then reactivated by incubation in 50 mM sodium phosphate buffer at pH 7 and 65°C. For all inactivation/reactivation steps a relation of 1/10 immobilized enzyme/solution (W/V) was used. The residual activity was always measured at pH 7 and 37°C, as previously described. When a constant value of residual activity was achieved, this was considered the maximum recovered activity. Three consecutive cycles of inactivation/reactivation of immobilized Gx-NOX were performed.

### Kinetic resolution of *rac*-1-phenylethanol

The oxidation of acetophenone was performed by the addition of an alcohol dehydrogenase from *Thermus thermophilus *HB27 (TtADH) immobilized on agarose activated with cyanogen bromide [41], and by an NAD^+ ^re-cycling system formed by NOX immobilized on Gx-ag. The reaction mixture contained 0.5 mM NAD^+^, 10 mM 1-phenylethanol and 200 μM FAD in 50 mM sodium phosphate at pH 7. The reaction was triggered at 55°C by adding 4 mg and 0.2 mg of immobilized TtADH and NOX respcetively. The reaction course was followed *via *reverse-phase HPLC (Spectra Physic SP 100 coupled with an UV detector Spectra Physic SP 8450) using a Kromasil C18 column (15 cm × 0.4 cm) supplied by Análisis Vínicos (Spain). The enantiomeric excess (*e.e*.) was determined by chiral reverse-phase HPLC, using pure commercial enantiomers as standards. The column was chiracel OD-R and the mobile phase was an isocratic mixture of 35% acetonitrile and 65% 10 mM sodium phosphate buffer at pH 7. The analyses were performed at fixed flow of 0.45 ml/min by recording the absorbance at 225 nm. 1-(R)-phenylethanol was eluted after 15.9 min while 1-(S)-phenylethanol was eluted at 17.4 min.

## Abbreviations

NOX: NADH-oxidase; PEI-ag: agarose 6BCL coated with polyethyleneimine; SD-ag: agarose 6BCL coated with dextran sulphate; Gx-ag: agarose 6BCL activate with glyoxyl groups; IDA-Cu^2+^-ag: agarose 6BCL activated with Cu^2+ ^coordinated with imidodiacetic acid; CNBr-ag: Agarose 6BCL activated with cyanogens bromide groups.

## Authors' contributions

JRM carried out the experimental work involving the biochemical analysis, protein purification and protein characterization and helped to JMB and CAG in their respective tasks. DV did all the cloning and protein expression work. JMB did the protein immobilization work. CAG did the reactivation experiments. AH and JB revised and participated in the experimental design that involved the molecular biology part. JMG and JB conceived the project and participated in the experimental design of the work. FLG coordinated the experiments and wrote the manuscript and supervised the experiments carried out by the other authors. JMG, JB and FLG carried out the revision of the manuscript. All the authors read and approved the final manuscript.

## Supplementary Material

Additional file 1 **Figure S1. Analysis SDS-PAGE of NOX purfication**. SDS-PAGE (12%) gels obtained during the sequential purification of NOX. Lanes: 1) Molecular weight markers; 3) Crude extract; 5) Supernatant after heat treatment at 80°C for 45 minutes; 6) Supernatant after incubation in presence of PEI agarose for 30 minutes; 8) Supernatant after incubation in the presence of sulfate-dextran agarose for 1 h. Click here for file

Additional file 2 **Table S1. H_2_O_2 _formation during NAD+ reduction by NOX**. The enzyme produces exclusively H_2_O_2 _as previously described Park et al. The fact that the recovery of H_2_O_2 _was sometimes less than 100% (70-80%), could be explained by the observation that the amount of H_2_O_2 _measured, was influenced by the time period between the NADH conversion and the actual measurement of H_2_O_2_. Apparently, the amount of H_2_O_2 _in the assay mixture slowly decreased, despite the absence of NADH, which was already completely converted at that moment. Click here for file

Additional file 3 **Figure S2. Alignment of NADH oxidase isolated from *Thermus thermophilus *HB27 and its counterpart isolated form *Thermus thermophilus *HB8**. Sequence HB27* resulted from cloning and sequencing of PCR product amplified from genomic DNA of *Thermus thermophilus *HB27 that we have at our laboratory. The sequence HB8 was corresponding to gene bank accession number: CAA42707.1. Both sequences were aligned using ClustalW algorithm. (*) identical residues. (:) different residues, highlighted in grey. Click here for file
