# Supplementary material for: New biotechnological perspectives of a NADH oxidase variant from Thermus thermophilus HB27 as NAD+-recycling enzyme
Source: BMC Biotechnol. 2011 Nov 3;11:101. doi: 10.1186/1472-6750-11-101 (PMC3238333; doi:10.1186/1472-6750-11-101)

Addtional file 1. Figure S1: Analysis SDS-PAGE of NOX purfication. SDS-PAGE (12 %) gels obtained during the sequential purification of NOX. Lanes: 1) Molecular weight markers; 3) Crude extract; 5) Supernatant after heat treatment at 80ºC for 45 minutes; 6) Supernatant after incubation in presence of PEI agarose for 30 minutes; 8) Supernatant after incubation in the presence of sulfate-dextran agarose for 1 h.

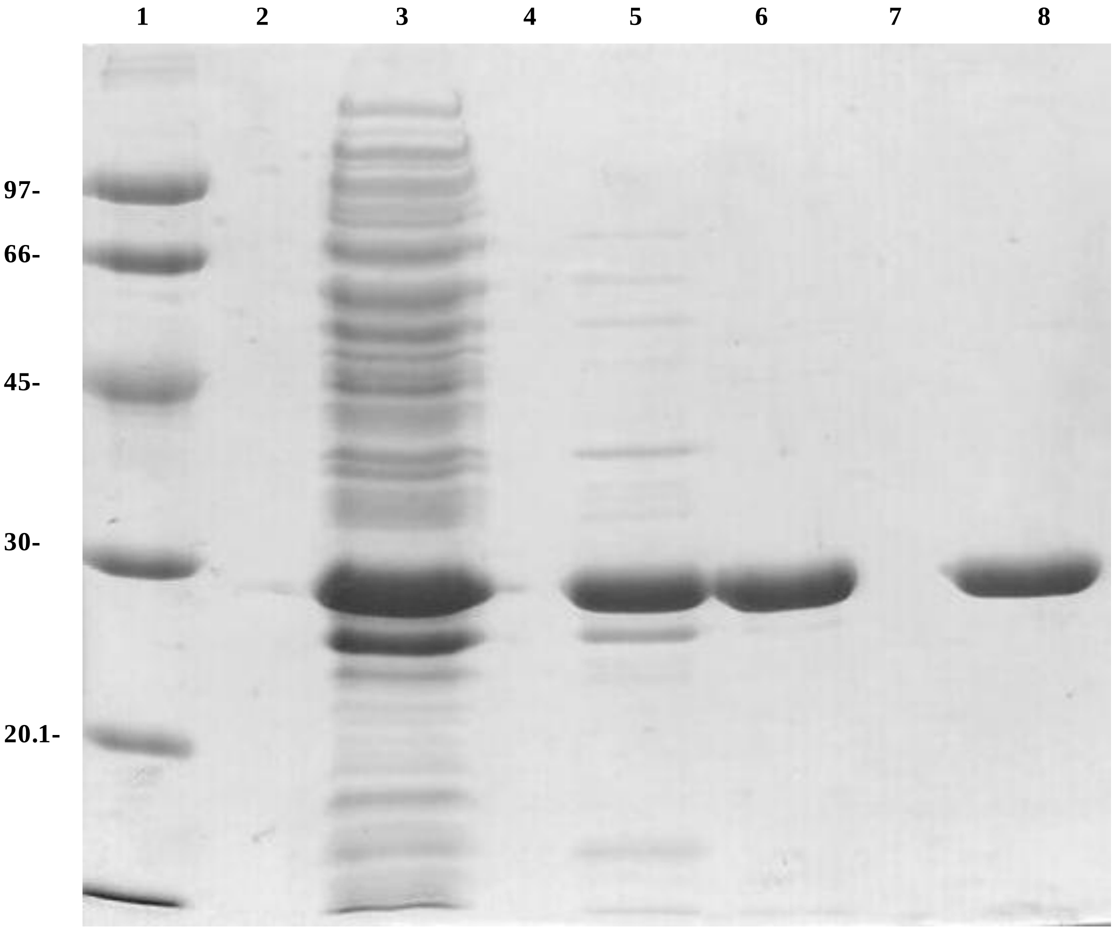

Supplement: Additional file 1 — Figure S1. Analysis SDS-PAGE of NOX purfication. SDS-PAGE (12%) gels obtained during the sequential purification of NOX. Lanes: 1) Molecular weight markers; 3) Crude extract; 5) Supernatant after heat treatment at 80°C for 45 minutes; 6) Supernatant after incubation in presence of PEI agarose for 30 minutes; 8) Supernatant after incubation in the presence of sulfate-dextran agarose for 1 h. [file 1472-6750-11-101-S1.PDF]
