# Supplementary material for: New biotechnological perspectives of a NADH oxidase variant from Thermus thermophilus HB27 as NAD+-recycling enzyme
Source: BMC Biotechnol. 2011 Nov 3;11:101. doi: 10.1186/1472-6750-11-101 (PMC3238333; doi:10.1186/1472-6750-11-101)
Supplement: Additional file 2 — Table S1. H2O2 formation during NAD+ reduction by NOX. The enzyme produces exclusively H2O2 as previously described Park et al. The fact that the recovery of H2O2 was sometimes less than 100% (70-80%), could be explained by the observation that the amount of H2O2 measured, was influenced by the time period between the NADH conversion and the actual measurement of H2O2. Apparently, the amount of H2O2 in the assay mixture slowly decreased, despite the absence of NADH, which was already completely converted at that moment. [file 1472-6750-11-101-S2.PDF]

Addtional file 2. Table S1. H_2O_2 formation during NAD^+ reduction by NOX. The H_2O_2 formed was measured as previously described [1].

NADH consumed (μM)	H_2O_2 formed (μM)	Ratio NADH/H_2O_2
8.14	6.77	0.83

The enzyme produces exclusively H_2O_2 as previously described Park et al.. The fact that the recovery of H_2O_2 was sometimes less than 100 % (70–80 %), could be explained by the observation that the amount of H_2O_2 measured, was influenced by the time period between the NADH conversion and the actual measurement of H_2O_2. Apparently, the amount of H_2O_2 in the assay mixture slowly decreased, despite the absence of NADH, which was already completely converted at that moment.

1. Kengen SWM, Van Der Oost J, De Vos WM: **Molecular characterization of H_2O_2-forming NADH oxidases from *Archaeogiobus fulgidus*.** *Eur J Biochem* 2003, **270:**2885-2894.
