# Supplementary material for: New biotechnological perspectives of a NADH oxidase variant from Thermus thermophilus HB27 as NAD+-recycling enzyme
Source: BMC Biotechnol. 2011 Nov 3;11:101. doi: 10.1186/1472-6750-11-101 (PMC3238333; doi:10.1186/1472-6750-11-101)
Supplement: Additional file 3 — Figure S2. Alignment of NADH oxidase isolated from Thermus thermophilus HB27 and its counterpart isolated form Thermus thermophilus HB8. Sequence HB27* resulted from cloning and sequencing of PCR product amplified from genomic DNA of Thermus thermophilus HB27 that we have at our laboratory. The sequence HB8 was corresponding to gene bank accession number: CAA42707.1. Both sequences were aligned using ClustalW algorithm. (*) identical residues. (:) different residues, highlighted in grey. [file 1472-6750-11-101-S3.PDF]

Additional file 3, Figure S2. Alignment of NADH oxidase isolated from *Thermus thermophilus* HB27 and its counterpart isolated form *Thermus thermophilus* HB8.

```
HB27*           MEATLPVLDAKTAALKRRSIRRYRKDPVPEGLLREILEAALRAPSAWNLQ
HB8             MEATLPVLDAKTAALKRRSIRRYRKDPVPEGLLREILEAALRAPSAWNLQ
                **************************************************

HB27*           PWRIVVVRDPATKRALREAAFGQAHVEEAPVVLVLYADLEDALAHLDEVI
HB8             PWRIVVVRDPATKRALREAAFGQAHVEEAPVVLVLYADLEDALAHLDEVI
                **************************************************

HB27*           HPGVQGERREAQKQAIQRAFAAMGQEARKAWASGQSYILLGYLLLLLEAY
HB8             HPGVQGERREAQKQAIQRAFAAMGQEARKAWASGQSYILLGYLLLLLEAY
                **************************************************

HB27*           GLGSVPMLGFDPERVKAILGLPSHAAIPALVALGYPAEEGYPSYRLPLER
HB8             GLGSVPMLGFDPERVRAILGLPSRAAIPALVALGYPAEEGYPSHRLPLER
                ***************:*******:*******************:******

HB27*           VVLWR
HB8             VVLWR
                *****
```

Sequence HB27* resulted from cloning and sequencing of PCR product amplified from genomic DNA of *Thermus thermophilus* HB27 that we have at our laboratory. The sequence HB8 was corresponding to gene bank accession number: **CAA42707.1.** Both sequences were aligned using ClustalW algorithm. (*) identical residues. (:) different residues, highlighted in grey.
